# The Book World of Medicine and Science

**Published:** 1899-12-02

**Authors:** 


### THE BOOK WORLD OF MEDICINE AND SCIENCE.

Twentieth Century Practice of Medicine. Edited by Thomas C. Stedman, M.D. Vols. XII. and XIII. (London: Sampson Low, Marston, and Co. Pp. 849, 621. 1897-8.)

The twelfth volume of this well-known encyclopædia is devoted to the study of mental diseases and of those of childhood and old age. The first category is divided into three parts, treating respectively of insanity, idiocy, and criminal anthropology, which are discussed by Dr. Blandford, Dr. Paul Sollier, and Professor Lombroso. Dr. Blandford's contribution is distinguished by his well-known grace of style and clearness of exposition; his subject is, however, obviously too great for the 250 pages allotted to it—probably all that could be spared in a work on general medicine—and the article consequently suffers on the side of sketchiness. The subject is taken up as if insanity were a single disease, the symptoms, diagnosis, pathology, and so forth being described in separate sections; the various forms are then treated of under the heading clinical varieties. It need hardly be said that the clinical portion of the essay is, as far as it goes, wholly admirable, but it must be admitted that the account of the pathology and morbid anatomy of insanity falls somewhat short of modern requirements. The article on idiocy is written by Dr. Sollier in a thoroughly scientific spirit. He distinguishes it as a symptom of an actual morbid affection of the nervous centres, while imbecility is merely the lowest form of general mental debility. In the matter of pathology, upon which he bases his classification, Dr. Sollier follows closely in the lines laid down by Bourneville, but his account of the symptoms is fresh and original, and derives particular value from the accuracy and suggestiveness of the psychological investigations upon which it is largely based. Especially fascinating are the sections relating to the pathological psychology of the emotions in idiocy.

The subject of criminal anthropology could hardly have been entrusted to a more distinguished authority than Professor Lombroso, and his account, although brief, is both sound and interesting. The summary of investigation into the bodily anomalies of more than six thousand criminals cannot fail to be instructive, as also are the observations on the cutaneous anæsthesia so frequently manifested by them. It is satisfactory to note that Professor Lombroso, unlike some of his extremist followers, does not desire to treat every criminal as a hospital or asylum patient; the prison plays its appropriate part in his scheme of the therapeutics of crime, and he still holds with capital punishment as the last resort.

Old age is treated of at considerable length by Dr. Boy-Tessier, of Marseilles. His essay is interesting from the charm of its style, which is unfortunately somewhat marred by inelegant translation; its practical value is naturally not very great.

The article on the diseases of children, by Dr. Jules Comby, is the least satisfactory in the book, and is, in fact, by no means worthy of its author's reputation. It is written rather in the style of an American "compend" than of an authoritative text-book; the therapeutical portion is, however, of considerable value.

In Volume XIII. is commenced the study of infectious diseases, prefaced by a series of articles touching the general pathology of infection. The first of these is from the pen of Professor Victor Vaughan, of Ann Arbor, and treats of ptomaïns, toxins, and leucomaïns. In connection with this subject, Professor Vaughan's reputation stands justly high, and his essay is a scholarly and well-digested summary of our present knowledge. In perusing it one is struck alike by the enormous amount of patient research which it records, and the comparative inadequacy of the results attained. With the isolation of the ptomaïns we seem to have come to a dead stop, and even if, as there is every hope, the chemical nature of toxins may soon be ascertained, we are almost no further along the road by which the action of these bodies upon protoplasm is to be explained. The solution of this problem is one of the ultimate goals of science, and we are confronted with the spectacle of the chemist and the pathologist standing on either side of a gulf which each is unable to bridge.

The important subject of infection and immunity has been allotted to Professor Ernst, of Harvard. The result has been somewhat unfortunate, particularly when it is remembered that the topic is at present the chosen battle-ground of opposing schools of pathologists. The article consists of a vast number of statements of fact jumbled together upon no ascertainable plan, and with an entire absence of epicriticism. There is no bibliography—a drawback also to Professor Vaughan's article—and the information is so far out of date that it is only charitable to suppose that Professor Ernst wrote the essay long before the time advertised on the title-page. As it is, Ehrlich's theory is not so much as mentioned, a fact which may fairly be admitted to absolve the critic from further discussion.

The general subject of waterborne disease receives ample attention from Dr. Solomon Smith and the late Mr. Ernest Hart. This is one of the best articles which have ever been written on this question; the combination of close reasoning and apt illustration with which it treats of typhoid and cholera should carry conviction even to an Indian Government official, and the articles on malaria, plumbism, and other affections are, if less full, equally cogent.

Dr. Dawson Williams writes of the duration of incubation and infectiousness, a subject upon which he is of course an authority. His article amplifies and extends the well-known and excellent report to the Clinical Society of London, for which he was in the first instance largely responsible.

Small-pox is discussed at length by Dr. J. W. Moore, of Dublin, in an article which deserves to become classical. Dr. Moore combines an extensive acquaintance with the work of others with an almost unrivalled personal experience and a ripe and temperate judgment. His account of the treatment of this eradicable scourge is unsurpassed in any work with which we are acquainted. Although Dr Moore has much to say of vaccination, vaccina is discussed by Dr. P. Brouardel, of Paris, whose article is, as one would expect, clear, scientific, and accurate; one misses with some surprise, however, any reference to the work of Seeley. The last article is from the pen of Dr. Jules Comby, who treats of mumps with a thoroughness in marked contrast to the style of his contribution to Vol. XII. It is needless to add that these two volumes are as handsomely produced as their predecessors, with which they form equally valuable contributions to medical literature.

